# Non-coding RNA regulation in pathogenic bacteria located inside eukaryotic cells

**DOI:** 10.3389/fcimb.2014.00162

**Published:** 2014-11-12

**Authors:** Álvaro D. Ortega, Juan J. Quereda, M. Graciela Pucciarelli, Francisco García-del Portillo

**Affiliations:** ^1^Centro Nacional de Biotecnología - Consejo Superior de Investigaciones Científicas (CNB-CSIC)Madrid, Spain; ^2^Departamento de Biología Molecular, Universidad Autónoma de Madrid, Centro de Biología Molecular ‘Severo Ochoa’ (CBMSO-CSIC)Madrid, Spain

**Keywords:** non-coding RNA, pathogen, intracellular infection, bacteria, regulation

## Abstract

Intracellular bacterial pathogens have evolved distinct lifestyles inside eukaryotic cells. Some pathogens coexist with the infected cell in an obligate intracellular state, whereas others transit between the extracellular and intracellular environment. Adaptation to these intracellular lifestyles is regulated in both space and time. Non-coding small RNAs (sRNAs) are post-transcriptional regulatory molecules that fine-tune important processes in bacterial physiology including cell envelope architecture, intermediate metabolism, bacterial communication, biofilm formation, and virulence. Recent studies have shown production of defined sRNA species by intracellular bacteria located inside eukaryotic cells. The molecules targeted by these sRNAs and their expression dynamics along the intracellular infection cycle remain, however, poorly characterized. Technical difficulties linked to the isolation of “intact” intracellular bacteria from infected host cells might explain why sRNA regulation in these specialized pathogens is still a largely unexplored field. Transition from the extracellular to the intracellular lifestyle provides an ideal scenario in which regulatory sRNAs are intended to participate; so much work must be done in this direction. This review focuses on sRNAs expressed by intracellular bacterial pathogens during the infection of eukaryotic cells, strategies used with these pathogens to identify sRNAs required for virulence, and the experimental technical challenges associated to this type of studies. We also discuss varied techniques for their potential application to study RNA regulation in intracellular bacterial infections.

## Introduction

Genome expression studies based on high-density tiling arrays and RNA deep sequencing (RNA-seq) show that a relatively high percentage of the bacterial genome is transcribed as non-coding RNA molecules (Gripenland et al., 2010; Sorek and Cossart, 2010; Lasa et al., 2012). These RNA molecules include antisense transcripts (asRNA) and small intergenic RNAs (sRNA). Many coding messenger RNAs are also transcribed with large stretches of untranslated RNA regions in their 5′ or 3′ ends, known as 5′-UTR and 3′-UTR. Non-coding RNAs regulate post-transcriptionally multiple processes in bacteria (Storz et al., 2011; Michaux et al., 2014). These molecules have been shown to control: (i) uptake and assimilation of nutrients (Sharma et al., 2011; Bobrovskyy and Vanderpool, 2013; Papenfort and Vogel, 2014); (ii) cell-to-cell communication (Lenz et al., 2004, 2005; Shao et al., 2013); (iii) envelope homeostasis (Papenfort et al., 2006; Song et al., 2008); (iv) biofilm formation (Ghaz-Jahanian et al., 2013; Shao et al., 2013; Zhao et al., 2013; Martinez and Vadyvaloo, 2014; Mika and Hengge, 2014); and (v) stress response to nutrient starvation, temperature or pH changes, iron limitation, oxygen deficiency, envelope alteration and oxidative damage (reviewed in Hoe et al., 2013). It is becoming evident that regulatory RNAs are also essential pieces in host-pathogen interactions. We refer the reader to excellent reviews describing virulence regulation by non-coding RNAs (Gripenland et al., 2010; Papenfort and Vogel, 2010; Caldelari et al., 2013; Harris et al., 2013).

In this review, we focus on the still limited knowledge related to the contribution of bacterial non-coding RNAs to the intracellular infection of eukaryotic cells. Despite the bulk of studies that have analyzed pathogens grown in laboratory conditions (Caldelari et al., 2013), much less work has been done in bacteria isolated from eukaryotic cells. This fact is emphasized here in the context of experimental challenges linked to studies with intracellular bacteria. We also highlight the necessity of analyzing material extracted from these bacteria to assess whether a particular non-coding RNA is required for establishing an intracellular infection. Representative non-coding RNAs characterized in pathogens isolated from eukaryotic cells are described. Lastly, we discuss the utility of current global expression techniques in RNA regulation studies involving intracellular bacterial pathogens.

## RNA regulation and the intracellular infection

The role of bacterial non-coding RNAs in virulence has received much attention in the last few years. Relevant data have been obtained in both cellular and animal models (Padalon-Brauch et al., 2008; Santiviago et al., 2009; Toledo-Arana et al., 2009; Gong et al., 2011; Mraheil et al., 2011; Ortega et al., 2012; Wurtzel et al., 2012; Gonzalo-Asensio et al., 2013; Yan et al., 2013; Warrier et al., 2014). Most of these studies focused on the identification of new sRNAs in bacterial pathogens; the genetic and molecular interactions occurring between a particular sRNA and well-defined effectors or virulence regulators; and, the phenotype of sRNA-defective mutants. In most cases, the lack of a single sRNA does not result in impaired fitness in the *in vivo* infection model. Unfortunately, the available information remains interspersed and, as such, the exact contribution that sRNA-mediated regulation has in the infection remains undefined. Critical aspects that future investigation could address are: (i) the identification of those sRNAs that are actually relevant for infection; (ii) the infection phase(s) at which their regulatory activity takes place; (iii) the mode by which the pathogen activates and de-activates these regulatory circuits; and (iv) the impact of sRNA activity in the infection process.

The first clear hint pointing at the involvement of sRNAs in infection was the discovery that mutants of varied bacterial pathogens lacking the RNA chaperone Hfq were attenuated in virulence (Roop et al., 2003; Sonnleitner et al., 2003; Christiansen et al., 2004; Ding et al., 2004; Sittka et al., 2007). Other global post-transcriptional RNA-binding proteins, like CsrA, control the transition between different physiological states in the infection process (Lucchetti-Miganeh et al., 2008). These studies demonstrated that de-regulation of sRNA-mediated control on gene expression leads to bacterial fitness defects in the host.

## Identifying non-coding RNAs in intracellular bacterial pathogens

Classical genetic screens in search for virulence factors missed sRNA loci probably due to their small size (50–250 nucleotide long) and/or mild phenotypes linked to their absence (Papenfort and Vogel, 2010). Transposon insertion sequencing, a technique first reported in 2009 that allows genome-wide analysis of insertions impairing bacterial fitness, was however suitable for identifying sRNA and other non-coding regulatory sequences (reviewed in Van Opijnen and Camilli, 2013). High-density transposon libraries identified non-coding RNAs important for growth of *Caulobacter crescentus* (Christen et al., 2011) and the pathogens *Mycobacterium tuberculosis* and *Streptococcus pneumoniae* (Mann et al., 2012; Zhang et al., 2012). Analysis of *S. pneumoniae* libraries in *in vivo* infection models also revealed sRNAs required for virulence (Mann et al., 2012). An important issue linked to these studies is to control that no factor besides the selection imposed by the experimental model affects insertion representativeness in the input and output bacterial pools. Usage of sufficiently large bacterial numbers, a critical parameter in studies dealing with intracellular bacteria (see below), might solve this population bottleneck. Phenotypic analyses can also be performed with small insertion pools having less representativeness than the minimal number of bacteria required to initiate infection in a particular infection model. To date, no genome-wide fitness study based on transposon insertion libraries has addressed the identification of non-coding RNAs in an intracellular infection model.

**Figure 1:**
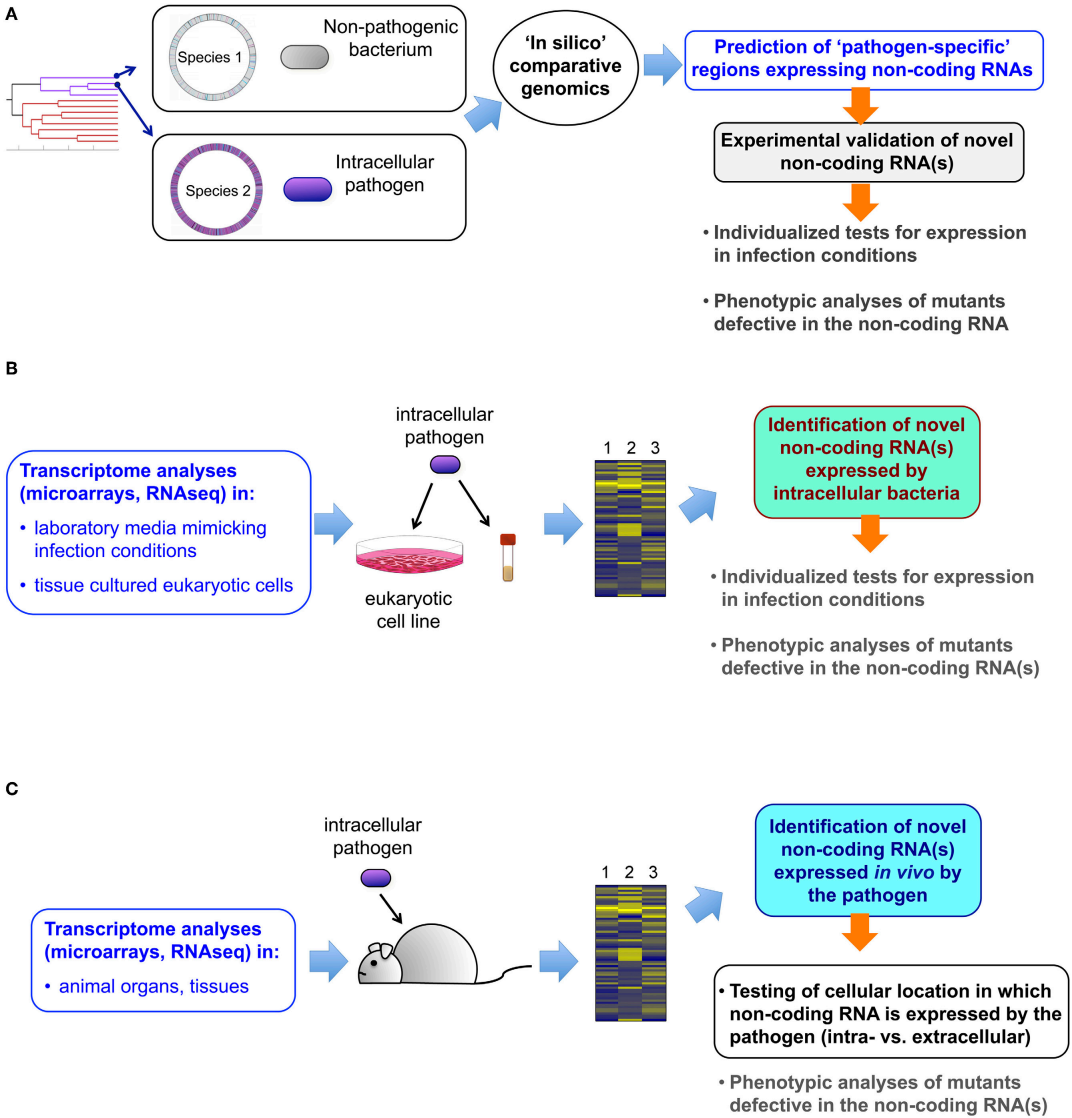
**Approaches leading to the identification of novel non-coding RNAs in intracellular bacterial pathogens. (A)** Comparative genomics of two closely and phylogenetically-related bacterial species, one pathogenic, and the other not, allows the identification of pathogen-specific genome regions predicted to generate non-coding RNA. These candidates can be further validated experimentally and tested for putative function in virulence with the corresponding defective mutants constructed *ad-hoc*; **(B)** analyses of transcriptomic data obtained with classical microarray technology or, alternatively by RNA-seq, allow the identification of non-coding RNAs in (i) the pathogen when growing in laboratory media mimicking infection conditions; or, (ii) in bacteria isolated from eukaryotic cells infected in *in vitro* using tissue cultured cells. These new non-coding RNAs are further validated experimentally and tested for putative role in virulence using defective mutants; **(C)** Transcriptomic data including information about non-coding RNA can also be obtained from the pathogen grown in tissues or organs of the animal. Although this approach does not provide a direct proof of production of the non-coding RNA in bacteria located inside the host cell, this possibility can be further tested in tissue culture cells. Experimental validation of the non-coding RNA and testing of its putative role in virulence are logical subsequent steps.

Distinct approaches that resulted in the successful identification of sRNAs are depicted in Figure 1. One of these, not restricted to intracellular bacterial pathogens, is the comparison of genomic regions that generate non-coding RNA in pathogenic and closely related non-pathogenic species (Padalon-Brauch et al., 2008; Wurtzel et al., 2012). The relevance of these analyses is supported by the existing link between the acquisition of foreign DNA and the emergence of new virulence traits (Dobrindt et al., 2004). Computational analyses allowed the identification of novel *Salmonella enterica* sRNA genes in “genetic islands” absent in the *Escherichia coli* genome, with two of these sRNAs, IsrJ, and IsrM, being involved in virulence (Padalon-Brauch et al., 2008; Gong et al., 2011) (Table 1). A genome-wide transcription start-site analysis performed in *Listeria monocytogenes* and *L. innocua* showed clear divergence between these two species in their “non-coding genome.” Among the 113 sRNAs identified, 25 were specific to *L. monocytogenes* (Wurtzel et al., 2012). Moreover, ~10% of the genes shared and expressed by these two *Listeria* species display different 5′-UTR lengths and 10 known *L. monocytogenes* virulence genes bear long 5′-UTR (Wurtzel et al., 2012). 5′-UTR in target mRNAs is the most common region where regulatory sRNAs bind. Altogether, these observations indicate that post-transcriptional regulation could play a relevant role in bacterial pathogens causing intracellular infections.

**Table 1:** **Non-coding RNAs of bacterial pathogens validated experimentally during the intracellular infection of eukaryotic cells^a^**.

**Intracellular pathogen**	**RNA^b^**	**Expression in intracellular vs. extracellular bacteria^c^**	**Target(s)^d^**	**Role in infection/phenotypes**	**References**
*Brucella abortus*	AbcR1 (sRNA)	Not tested individually for expression inside eukaryotic cells	BAB1_1794, BAB2_0612	Lack of both RNAs *impairs* intramacrophage survival and virulence in mice.	Caswell et al., 2012
	AbcR2 (asRNA)				
*Coxiella burnetii*	CbsR1, CbsR2, CbsR3, CbsR4, CbsR9, CbsR11, CbsR12, CbsR14 (sRNAs)	Up	Unknown	Unknown	Warrier et al., 2014
*C. burnetii*	SsrS (6S)	Up	RNA-polymerase	Unknown	Warrier et al., 2014
*Chlamydia penumoniae*	CPIG0142, CPIG0207, CPIG0294, CPIG0397, CPIG0457, CPIG0564, CPIG0692, CPIG0953, CPIG0954 (sRNAs)	Obligate intracellular	Unknown	Unknown	Albrecht et al., 2011
*Chlamydia penumoniae*	CPAS0152, CPAS0294 (asRNA), CPS0457 (sense overlapping RNA)	Obligate intracellular	Unknown	Unknown	Albrecht et al., 2011
*Chlamydia thrachomatis, C. caviae, C. pneumoniae, C. muridarum*	IhtA (sRNA)	Up	Histone homolog Hc1	Required for RB development cycle by inhibiting translation of Hc1, a chromatin condensing protein present in EB forms.	Grieshaber et al., 2006; Albrecht et al., 2011; Tattersall et al., 2012
*C. thrachomatis*	CtrR1, CtrR2, CtrR3, CtrR4, CtrR5, CtrR6, CtrR7, CtrR8, CtrR0332, pL2-sRNA1 (sRNAs)	Obligate intracellular	(many predicted)	Unknown	Albrecht et al., 2010
*Francisella turalensis*	FtrA, FtrB (sRNAs)	Not tested individually for expression inside eukaryotic cells	Unknown	Not required for intramacrophage proliferation or for causing disease in mice.	Postic et al., 2010
*F. tularensis*	FtrC (sRNA)	Not tested individually for expression inside eukaryotic cells	*FTL_1293*	Attenuates virulence and impair intracellular proliferation if overproduced.	Postic et al., 2012
*Legionella pneumophila*	SsrS (6S)	Not tested individually for expression inside eukaryotic cells	RNA-polymerase	Required for intramacrophage proliferation.	Faucher et al., 2010
*Listeria monocytogenes*	Rli27 (sRNA)	Up	*lmo0514*	Unknown	Quereda et al., 2014
*L. monocytogenes*	Rli31, Rli33-1 (sRNAs)	Up	*pgdA, pbpX* (Rli31) unknown (Rli33-1)	Survival inside macrophages/attenuation in the mice and the *Galleria mellonella* insect model.	Mraheil et al., 2011; Burke et al., 2014
*L. monocytogenes*	Rli50, Rli112 (sRNAs)	Unaltered	Unknown	Survival inside macrophages/attenuation in the mice and the *Galleria mellonella* insect model.	Mraheil et al., 2011
*L. monocytogenes*	LhrC	Up (transcriptome)	adhesin LapB	Unknown	Mraheil et al., 2011; Sievers et al., 2014
*L. monocytogenes*	Rli55	Up (transcriptome)			Mraheil et al., 2011
*L. monocytogenes*	anti0055 (asRNA)	Up	*purA (lmo0055)*	Unknown	Behrens et al., 2014
*L. monocytogenes*	anti2106 (asRNA)	Up	*lmo2106*	Unknown	Behrens et al., 2014
*L. monocytogenes*	anti2225 (asRNA)	Up	*fumC (lmo2225)*	Unknown	Behrens et al., 2014
*L. monocytogenes*	anti2330 (asRNA)	Down	*lmo2331*	Unknown	Behrens et al., 2014
*L. monocytogenes*	anti2367 (asRNA)	Up	*pgi (lmo2367)*	Unknown	Behrens et al., 2014
*Mycobacterium tuberculosis*	AsDes (asRNA)	Not tested individually inside eukaryotic cells	*desA1*	Unknown	Arnvig and Young, 2009
*Salmonella enterica* serovar Typhimurium (*S*. Typhimurium)	RyhB-1 (sRNA)	Up	*cyoABC, cydB, cybC, nirBCD*	Increased sensitivity to nitrosilating and oxidative agents.	Padalon-Brauch et al., 2008; Ortega et al., 2012; Calderon et al., 2014a,b;
*S.* Typhimurium	RyhB-2 (asRNA)	Up	*yeaQ, cyoABC, cydB, cybC, nirBCD*	Increased sensitivity to nitrosilating and oxidative agents.	Padalon-Brauch et al., 2008; Ortega et al., 2012; Calderon et al., 2014a,b;
*S.* Typhimurium	IsrA, rseX, IstR-2, IsrG, T44, IsrK (sRNAs)	Up	Unknown	Unknown	Ortega et al., 2012
*S.* Typhimurium	OxyS, IsrB, IsrE, IsrF, IsrJ, IsrK, IsrM, IsrN, IsrO, IsrP, IsrQ (sRNAs)	Up	Unknown	Unknown	Padalon-Brauch et al., 2008
*S.* Typhimurium	IsrC (asRNA)	Up	*msgA*	Unknown	Padalon-Brauch et al., 2008
*S.* Typhimurium	InvR (sRNA)	Up	*ompD*	Unknown	Pfeiffer et al., 2007; Ortega et al., 2012
*S.* Typhimurium	IsrH-1, IsrI (sRNAs)	Up (macrophages), Down (fibroblasts)	Unknown	Unknown	Padalon-Brauch et al., 2008; Ortega et al., 2012
*S.* Typhimurium	SraL (sRNA)	Down	TF (trigger factor)	Unknown	Ortega et al., 2012; Silva et al., 2013
*S.* Typhimurium	MicC, CyaR (sRNAs)	Unaltered	Unknown	Unknown	Ortega et al., 2012
*S.* Typhimurium	GlmZ, SroC, IsrH-1, DsrA, RydC, IsrI (sRNAs), SsrS (6S)	Down	Unknown	Unknown	Ortega et al., 2012
*S.* Typhimurium	IesR-1 (asRNA)	Up	PSLT047	Unknown	Gonzalo-Asensio et al., 2013
*S.* Typhimurium	IsrM (sRNA)	Up (in mouse organs). Not tested individually for expression inside eukaryotic cells	*hilE, sopA*	Required for invasion and intracellular proliferation inside macrophages.	Gong et al., 2011

a Validated at least by one of the following methods: cDNA cloning, Northern blot, and/or strand-specific qRT-PCR.

b asRNA, antisense RNA; sRNA, small RNA encoded in intergenic regions; 6S, RNAP-binding 6S RNA.

c Those non-coding RNAs not tested individually for expression in intracellular bacteria are also indicated.

d Only validated targets are indicated.

*In vitro* tissue culture infections have also been exploited to determine the sRNAome in intracellular bacteria (Table 1). Twenty-nine regulatory RNAs, including non-coding antisense asRNAs, were detected by RNA-seq in *L. monocytogenes* isolated from murine macrophages (Mraheil et al., 2011). Among these, Rli31, Rli33-1, and Rli50 regulate intracellular growth in macrophages. Mutants lacking any of these sRNAs are attenuated in the mouse and insect infection models (Mraheil et al., 2011) (Table 1). Rli31 was recently shown to regulate enzymes involved in modification of peptidoglycan structure (Burke et al., 2014). A deeper RNA-seq analysis performed with the SOLiD platform on size-fractioned RNA obtained from intracellular *L. monocytogenes* uncovered nine novel asRNAs (Behrens et al., 2014). anti2367, which maps opposite to the gene *lmo2367* encoding a glucose-6-phosphate isomerase, was detected exclusively in intracellular bacteria (Behrens et al., 2014). *lmo2367* down-regulation raises interesting questions about the relevance of non-coding RNA regulation in carbon metabolism of intracellular bacteria. RNA-seq performed in the intracellular pathogen *Coxiella burnetii*, which alternates between a metabolically-active large cell variant (LCV) and a dormant small cell variant (SCV), identified 15 sRNAs in bacteria isolated from eukaryotic cells and axenic cultures (Warrier et al., 2014) (Table 1). Most of these sRNAs show differential expression, with increased levels in LCVs compared to bacteria grown in axenic conditions (Warrier et al., 2014). Deep-sequencing performed in the *Legionella pneumophila*-amoeba infection model uncovered 70 novel sRNAs in this pathogen, with some expressed preferentially during infection (Weissenmayer et al., 2011). Genome-wide expression profiling was also obtained in non-growing *S. enterica* serovar Typhimurium (S. Typhimurium) isolated from fibroblasts (Ortega et al., 2012; Gonzalo-Asensio et al., 2013; Nunez-Hernandez et al., 2013). Intracellular bacteria up-regulate the sRNAs RyhB-1, IstR-2, and RseX, together with the *Samonella*-specific sRNAs IsrA, IsrG, and RyhB-2 (Ortega et al., 2012) (Table 1). This study also identified a novel *S. Typhimurium*-specific non-coding RNA termed IesR-1 for “intracellular expressed sRNA”-1. IesR-1 expression is undetectable in bacteria grown in laboratory media, including those reported to induce virulence genes, and in bacteria growing inside epithelial cells. Noteworthy, IesR-1 expression exhibits a marked increase (~200-fold) in non-growing intracellular bacteria (Gonzalo-Asensio et al., 2013) (Table 1). These findings indicate that some intracellular bacterial pathogens might exploit sRNA regulation to restrain intracellular growth and persist within the host cell.

Another area of active research involves the identification and functional analysis of sRNAs based on *in vivo* infection models. In a reference study, a pool of *Salmonella* deletion mutants covering 1023 genes was injected into mice and the representation of each mutant compared in the spleen and the input pool (Santiviago et al., 2009). Mutants in the sRNAs IstR, OxyS, and SroA were affected and further tested against wild type bacteria in individual competition assays. These mutants displayed small, but reproducible, phenotypes of virulence attenuation with the only exception of the *oxyS* mutant, which exhibited increased fitness (Santiviago et al., 2009). In other studies, bacteria were isolated from organs of infected animals and their transcriptome compared to the obtained in bacteria grown *in vitro* (Toledo-Arana et al., 2009; Arnvig et al., 2011; Yan et al., 2013). The sRNAome of *Yersinia pestis* grown *in vitro* and in the lungs of mice was determined by RNA-seq (Yan et al., 2013). One hundred-four sRNAs were identified, of which 78 were novel sRNAs including 62 intergenic and 16 antisense asRNAs. *Y. pestis* growing in the lungs induce CyaR, 6S RNA, RyhB-1, RyhB-2, RybB, and sR039 compared to *in vitro*-grown bacteria. RNA-seq was also used in samples obtained from lungs of mice chronically infected with *M. tuberculosis* (Arnvig et al., 2011). The sRNAs MTS2823, MTS0997, and MTS1338 are abundant in stationary phase bacteria but accumulate to even higher levels in bacteria located in the lungs of chronically infected mice, supporting a role in the infection. Tiling arrays were used to analyze the transcriptional landscape of *L. monocytogenes* in infection relevant conditions (Toledo-Arana et al., 2009). RNA expression profiles were obtained from bacteria grown *in vitro*, isolated from the intestinal lumen of infected mice and grown *ex vivo* in human blood. Fifty sRNAs were discovered, 29 of which were novel, including asRNAs covering several open-reading frames and long overlapping 5′-UTRs and 3′-UTRs (Toledo-Arana et al., 2009). These studies also showed an extensive gene expression reshaping in *L. monocytogenes* isolated from the intestines compared to bacteria growing in blood or laboratory medium. Twelve sRNAs are induced in the intestine and 16 in blood. Rli40, Rli29, Rli27, Rli22, and RliB are induced in both host environments, suggesting a regulatory role for these sRNAs in the switch from saprophytism to intracellular parasitism. Most of these sRNAs have not yet been studied in bacteria directly isolated from eukaryotic cells (Table 1).

## Dynamics of non-coding RNA regulation along the infection

A transcriptional map depicting transcription start sites, non-translated regulatory regions, and expression profiles of bacterial pathogens in different growth conditions is an invaluable resource. However, it just reflects a snapshot in infection biology. To have a realistic image of regulatory circuits controlled by non-coding RNAs along the infection, the variable “time” should be brought into play. sRNA expression and activity change over time when bacteria grow in laboratory media (Chao et al., 2012; Kroger et al., 2012). This is especially relevant in stress conditions, in which sRNA-mediated post-transcriptional regulation is most prominent (Kroger et al., 2013; Stubben et al., 2014). *In vivo*, the infection is a complex multi-step process in which the pathogen copes with diverse stresses imposed by host defenses. Thus, foodborne enteric pathogens face stomach acid pH, bile salts, antimicrobial peptides, local inflammation, and competition against resident microbiota. Adaptation to these different niches entails dynamic gene expression changes, which means that there is a stage-dependent readjustment of a preexisting transcriptome. Gene expression analyses must also consider the reference sample, which might bias data interpretation. As a representative example, the *S.* Typhimurium sRNA SraL is expressed at higher levels by non-growing intracellular bacteria than by actively growing extracellular bacteria (Ortega et al., 2012). This observation might indicate that this sRNA plays a role during infection. However, SraL is barely expressed by actively growing bacteria although induced at stationary phase in an RpoS-dependent manner (Silva et al., 2013). Bacteria used to infect eukaryotic cells are normally grown to stationary phase. Noteworthy, SraL levels do not increase anymore upon bacterial entry into fibroblasts and decline progressively along the infection (Ortega et al., 2012). Unless a bacterial sample “prior to infection” is selected as reference, or a time course along the infection is performed, SraL will not be identified as a carryover sRNA that derives from a previous stage because has probably a long half-life. Dynamic gene expression analyses are therefore critical for characterizing sRNA function.

Conventional genetic approaches do not usually provide key information to define sRNA function. As aforementioned, RyhB-1 and RyhB-2 levels increase in intracellular *S.* Typhimurium and in *Y. pestis* isolated from infected mice lungs. However, single and double mutant strains lacking these two sRNAs do not show a profound virulence defect *in vivo* (Ortega et al., 2012; Yan et al., 2013). This might be explained by functional redundancy and/or an efficient adaptation. To overcome this issue, most studies rely on pulsed ectopic expression of the sRNA in bacteria grown in laboratory media to experimentally identify sRNA targets. This approach should not be formally acceptable when the aim is to characterize the regulatory role (i.e., the identification of the cellular targets) of an sRNA during infection. As abovementioned, transcriptome reshapes in infecting bacteria (intracellular, intestinal, etc.) when compared to bacteria grown in axenic cultures (Toledo-Arana et al., 2009; Wurtzel et al., 2012; Nunez-Hernandez et al., 2013). So, using bacteria growing in a flask for a pulsed expression of an sRNA up-regulated intracellularly might be misleading. Unfortunately, there is not an easy technical solution to this caveat. An approach could be to perform RNA-seq in wild-type and sRNA-defective bacteria isolated from eukaryotic cells. Target identification also relies on the definition of total protein content (proteome) in the presence or absence of the sRNA. A few studies have shown that such analyses are feasible in intracellular bacteria isolated from eukaryotic cells (see below).

An additional important issue to consider is that most known trans-acting sRNAs regulate a plethora of targets by degenerate interaction. This regulation is superimposed to other layers of gene expression regulation (transcription factors, RNAses, sRNAs), which contribute to build up the transcriptome at a precise time. An ectopically-expressed sRNA will be just able to bind mRNAs that are already “there.” In order to identify a specific sRNA target relevant for infection, the sRNA and the target should be both co-expressed in time and space, which might not be the case for bacteria growing in laboratory media. The ectopic induction of the sRNA might also lead to “off-target” effects on mRNAs that would never be naturally co-expressed with the sRNA in the infection scenario. These observations indicate that the most rational way to get functionally relevant information from regulators is to determine both global gene expression and proteome dynamics along the infection. Proteomic analyses performed in bacteria isolated from cultured eukaryotic cells are known for *L. monocytogenes* (Donaldson et al., 2011; Garcia-Del Portillo et al., 2011), *S.* Typhimurium (Shi et al., 2006), and *Staphylococcus aureus* (Surmann et al., 2014). Pathogen proteome has also determined *in vivo* using engineered *S.* Typhimurium, expressing fluorescent protein and sorted using organ extracts obtained from infected mice (Bumann, 2010). Although *in vivo* studies provide useful information on proteins produced by the pathogen in the animal, they do not differentiate among those proteins produced inside host cells from those that the pathogen may eventually synthetize in extracellular locations. More recent reports highlight the capacity of new technologies to accomplish quantitative proteomic analysis from as low as 10^6^ internalized bacteria using cultured eukaryotic cells (Pfortner et al., 2013). Therefore, the combination of RNAome and proteome data seems feasible in a near future, with the possibility of experimentally testing targets of non-coding RNAs directly in bacteria isolated from infected eukaryotic cells.

## Examples of regulation by non-coding RNAs in intracellular bacteria

**Figure 2:**
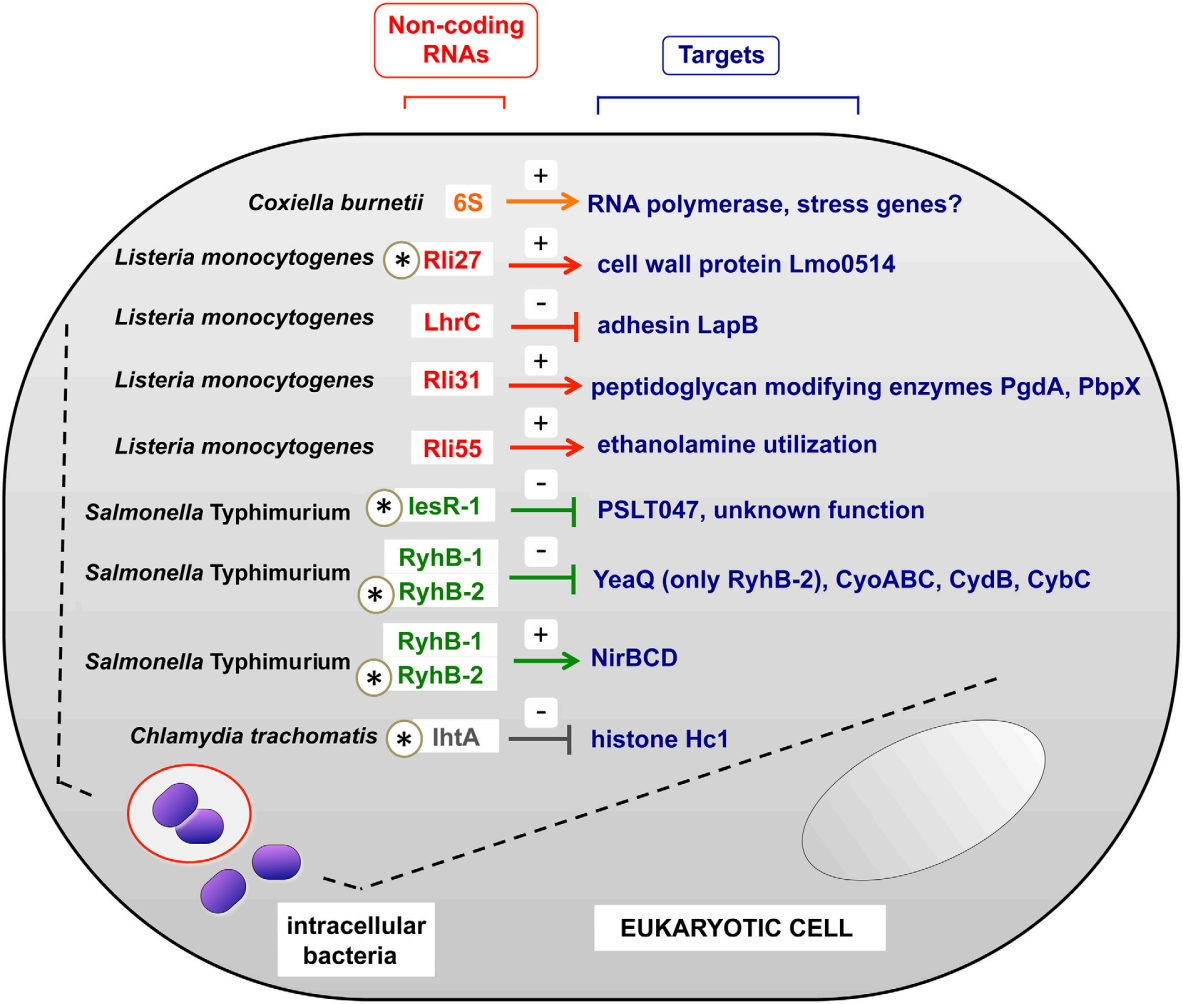
**Examples of sRNAs produced by intracellular bacterial pathogens inside the host eukaryotic cell**. All sRNAs shown here were validated experimentally in bacteria isolated from eukaryotic cells. Note the existence of negative and positive regulation, indicated with (−) and (+) signs respectively. In those sRNAs indicated with an asterisk, the regulation over the indicated target has been demonstrated in bacteria residing within the infected eukaryotic cell. For the case of RyhB-2 in *S.* Typhimurium, only the regulation over *yeaQ* has been proved to occur in intracellular bacteria. Intracellular bacteria are shown in phagosomal and cytosolic locations, covering the different lifestyles of the distinct pathogens shown. See also Table 1 for details.

In this section we refer to sRNAs studied directly in intracellular bacterial pathogens obtained from infected eukaryotic cells. Table 1 summarizes in a comprehensive manner non-coding RNAs characterized in these experimental conditions. Some representative examples, for which the target(s) has been identified, are also highlighted in Figure 2.

*L. pneumophila* expresses the non-coding 6S RNA at much higher levels inside macrophages than in laboratory media (Faucher et al., 2010). 6S RNA regulates positively genes encoding effector proteins translocated by a type IV secretion system and genes encoding proteins involved in nutrient acquisition and stress adaptation. Regulation exerted by 6S RNA on these targets was studied in post-exponential growth phase, thought to mimic late stages of the intracellular infection cycle (Hayashi et al., 2010). Noteworthy, lack of 6S RNA diminishes ~10-fold *L. pneumophila* intracellular growth in both protozoan and mammalian host cells with no detectable effect on extracellular bacteria growing in laboratory media (Faucher et al., 2010). 6S RNA therefore exemplifies a regulator necessary for optimal bacterial growth within host cells. Like *L. pneumophila*, the intracellular pathogen *C. burnetii* increases 6S RNA relative levels inside eukaryotic cells compared to axenic cultures (Warrier et al., 2014). *C. burnetii* 6S RNA specifically binds to RNA polymerase like its *E. coli* homolog, which interacts with and sequesters RNA polymerase σ^70^ subunit to allow stress genes transcription by σ^S^ (Wassarman and Storz, 2000).

Recent work in intracellular *L. monocytogenes* has linked sRNA regulation with cell wall-associated proteins. The sRNA Rli27 is induced ~20–25 fold in intracellular bacteria compared to bacteria grown in laboratory media (Quereda et al., 2014). Rli27 acts *in trans* by targeting a long (234-nt) 5′-UTR of the *lmo0514* gene, which encodes a surface protein of unknown function that is abundant in the cell wall of intracellular bacteria (Garcia-Del Portillo et al., 2011). Interestingly, the “long” *lmo0514* transcript variant that is also induced by intracellular bacteria bears an Rli27-binding region in its 234 nt 5′-UTR. The interaction between Rli27 and the long 5′-UTR of *lmo0514* is predicted to open the Shine-Dalgarno site to facilitate Lmo0514 protein translation (Quereda et al., 2014). Rli27 has also been claimed to regulate in *cis lmo0412*, a gene encoding a membrane protein required for virulence (Quereda and Pucciarelli, 2014). Another recent study reported that the multicopy sRNA LhrC is induced by *L. monocytogenes* in the intracellular environment and in response to envelope stress (Sievers et al., 2014). LhrC targets *in trans* and impedes the translation of *lapB* mRNA, which encodes an adhesin required for bacterial entry into mammalian cells and for virulence (Sievers et al., 2014). LhrC might be exploited by the pathogen to down-regulate an adhesin following the colonization of the intracellular niche. *L. monocytogenes* virulence is also regulated by Rli55, an sRNA detected in intra- and extracellular bacteria (Mraheil et al., 2011) and whose activity is modulated by a vitamin B12-binding riboswitch (Mellin et al., 2014). Rli55 controls expression of the ethanolamine utilization pathway genes by sequestering the two-component response regulator EutV through a binding-site located within the RNA. *L. monocytogenes* mutants defective in ethanolamine utilization, or in its regulation by Rli55, are markedly attenuated in the mouse infection model (Mellin et al., 2014).

As abovementioned, IesR-1 is an *S.* Typhimurium sRNA up-regulated by non-growing bacteria persisting inside fibroblasts (Gonzalo-Asensio et al., 2013). IesR-1, which is encoded by the pSLT virulence plasmid, overlaps and is complementary to the flanking gene *PSLT047*, of unknown function. IesR-1 is proposed to regulate this target by acting as an antisense RNA over the *PSLT047* mRNA. An interaction at the respective 3′ ends of these RNAs might explain the diminished PSLT047 protein levels observed in intracellular bacteria. IesR-1 deletion results in decreased capacity of *S.* Typhimurium to persist within human fibroblasts and impaired virulence attenuation in the mouse typhoid model (Gonzalo-Asensio et al., 2013).

Another example of RNA regulation inside eukaryotic cells is found with the obligate intracellular pathogen *Chlamydia trachomatis*. This pathogen uses during its life cycle the sRNA IhtA to control differentiation of reticulate bodies (RBs, the metabolically active and replicate form) to elementary bodies (EBs, the extracellular and metabolically inactive form). Two chlamydial histone H1 homologs; Hc1, encoded by the gene *hctA*, and Hc2, bind to and compact the bacterial chromosome making the EBs transcriptionally and translationally inactive. IhtA (for inhibitor of *hctA* translation) is only expressed in RBs where it inhibits *hctA* translation without affecting *hctA* mRNA transcription or stability. Conversely, Hc1 is only present in purified EBs where it densely compacts the chromosome, rendering *Chlamydia* in a metabolically inert stage (Grieshaber et al., 2006).

## Isolating intracellular bacteria from eukaryotic cells

Infection of cultured eukaryotic cells has been widely used to characterize major virulence factors in intracellular bacteria, their cellular targets and the underlying signaling pathways (Cossart et al., 2005). In this infection model, intracellular bacteria can be physically separated from the eukaryotic cell to perform biochemical, genetic and cell biology studies. This is a major issue when attempting to identify novel sRNAs important for the intracellular infection. Nonetheless, the *in vitro* infection model imposes a careful control of experimental conditions and a clear definition of objectives to be addressed (Stacey, 2012). Thus, host cell types naturally targeted by the intracellular pathogen in the animal should ideally be used. Mammalian cell lines as Caco-2 (enterocyte-like cells from colorectal adenocarcinoma) and Int-407 (derived from human embryonic jejunum and ileum) are commonly used in studies with enteric pathogens. Other cell lines as JEG-3 (human choriocarcinoma) or HIBCPP cells (malignant choroids plexus papilloma cell line), are preferred for studying pathogens that target the female reproductive system or cross the blood-cerebrospinal fluid (CSF) barrier, respectively (Stacey, 2012). On the technical side, scalability of infected cultures is often essential to obtain the required “mass” of intracellular bacteria. This parameter is especially critical when intracellular bacteria enter into a dormancy-like state and the average bacterial number per infected cell is relatively low (Garcia-Del Portillo et al., 2008). Other factors in the eukaryotic cell culture as population size, local cell density or location in a cell islet edge, influence the infection outcome (Snijder et al., 2009). The intrinsic heterogeneity of the model, in which not all the cultured cells become infected and the number and location of bacteria differ among infected cells, is also an important parameter to consider (Garcia-Del Portillo, 2008). Most studies performed to date deal with “average” data referring to host and pathogen responses. A way to avoid this is to sort populations of infected vs. non-infected cells derived from the same culture. Low yields and the inherent cell manipulation might explain why studies based on sorting procedures are scarce (Schulte et al., 2011). Novel technologies developed for transcriptomic and proteomic analyses of single eukaryotic or bacterial cells could overcome some of these technical caveats (Kang et al., 2011; Tang et al., 2011; Hughes et al., 2014). Thus, RNA-seq can be applied to single cells that are isolated using fluorescence-activated cell sorting, optofluidics, microfluidics, or laser-capture tissue micro-dissection (Saliba et al., 2014). Single-cell RNA-seq is useful to detect cell-to-cell variability in non-coding RNA expression. Physiology of intracellular bacteria located in defined subcellular locations can be also monitored with reporters based on fluorescent proteins expressed constitutively or promoters responding to the infection (Campbell-Valois and Sansonetti, 2014). Transcriptome and proteome quantification with single-molecule sensitivity was performed in single *E. coli* cells (Taniguchi et al., 2010). This study used a chromosomal YFP fusion library composed of >1000 strains, each of them tagged with a YFP sequence in the gene of interest. Strains are monitored at the microscope for the YFP signal and simultaneously in a fluorescent *in situ* hybridization (FISH) assay that uses a common oligonucleotide probe to the *yfp* sequence. Signals are recorded on a microfluidic chamber in which each strain is grown in independent cells (Taniguchi et al., 2010). Adaptation of this experimental set-up to an *in vitro* infection model of cultured eukaryotic cells seems in principle affordable. The chromosomal YFP fusion library can be constructed in wild-type and sRNA-null genetic backgrounds, with the possibility of testing a third library consisting in bacteria that overexpress the sRNA of interest. The design could also consider using a compatible fluorophore-conjugated oligonucleotide that binds to the sRNA itself. Thus, protein and transcript levels of the target, determined from YFP and the *ypf* probe signals respectively, could be correlated at any time in the experiment to a defined amount of the regulator deduced from the signal of the sRNA-specific probe. Like the screenings based on interference RNA, high-throughput technologies can scale experimental conditions for infecting cultured eukaryotic cells with these bacterial libraries using microfluidic chambers. A probable caveat to this type of approach relies in the difficulty for generating the chromosomal fusion libraries. Although some intracellular bacterial pathogens as *S. enterica* are manipulable genetically others, as some obligate intracellular pathogens, are not. Additional factors to evaluate are: (i) the limitation that the genetic procedure has for analyzing non-essential genes or essential genes whose function is not perturbed by the tag; and, (ii) the possibility that tagging might affect function in protein(s) contributing to the infection. Nonetheless, these factors influence any type of screening and they should not impact negatively the benefits that these massive analyses might have for studying RNA regulation in intracellular bacterial pathogens.

Another key technical issue of the *in vitro* infection model is the multiplicity of infection (MOI), a ratio often used disparately by different labs even for the same cell line and pathogen. An optimal adjustment of this parameter prevents over-infection and lysis of heavily-infected cells due to massive bacterial load (Francis and Thomas, 1996). In some cases, such lysis is triggered by the action of cytotoxic factors as pore-forming or membrane-disrupting toxins secreted to the cell culture medium by the pathogen (Kuhbacher et al., 2014). A careful control of these biological and technical facts and the accomplishment, when affordable, of single-cell assays, might pave the way to the identification of novel non-coding RNAs produced by bacterial pathogens inside eukaryotic cells.

## Studying gene expression in intracellular bacteria

As discussed in previous section, some technologies allow to monitoring expression of single molecules (RNA, protein) in individual bacteria. However, to date most of those studies have been performed in bacteria grown in axenic cultures and it is not trivial to extend such advances to an *in vitro* infection model in which intracellular bacterial pathogens not manipulable genetically are used. As an alternative, there are methods that record gene expression in intact, non-manipulated, living cells. That is the case of the SmartFlare™ technology, based on oligonucleotide probes attached to gold particles that are endocytosed by the eukaryotic cell (Seferos et al., 2007). These particles carry a “reporter” and a “capture” probe, with the reporter probe conjugated to a fluorophore that is quenched by the gold particle. This fluorophore become active when the “reporter” probe is released from the gold particle following replacement by the mRNA of interest, whose expression level is then directly monitored at a function of time. Ideally, this approach could be applied to monitor gene expression in intracellular bacteria. However, SmartFlare™ probes remain in the cytosol and do not pass through the nuclear membrane, which indicate they might not penetrate the bacterial envelope. To our knowledge, the potential utilization of these probes in bacteria has not been tested yet. Alternatively, SmartFlare™ technology, which allows visualization of the fluorophore signal in individual living cells, could be useful to register changes in host gene expression in infected and uninfected cells. A challenge here is to select the appropriate host genes responding to the intracellular bacterial infection. An approach directed to first determine the transcriptome of infected cells seems preferable. This prior analysis could provide a list of genes displaying expression profiles appropriate to later monitor in real-time along the infection.

Gene expression can also be analyzed in bacteria isolated from infected host cells. The field rapidly expanded following optimization of new methods to purify RNA from intracellular bacteria. In 2003, a method was described involving selective lysis of infected eukaryotic cells in a solution containing 0.1% SDS, 1% (v/v) acidic phenol, and 19% (v/v) ethanol (Eriksson et al., 2003). Incubation in this solution ensured bacterial RNA integrity and allowed physical separation of intact intracellular bacteria from debris of the infected eukaryotic cells. Adjustment of the detergent concentration was later shown to be required for optimal results depending on the host cell type, e.g., 0.1% SDS is sufficient in the *Salmonella*-macrophage infection model whereas 0.4% SDS is needed to lyse efficiently fibroblasts infected with this pathogen (Ortega et al., 2012; Gonzalo-Asensio et al., 2013; Nunez-Hernandez et al., 2013). Despite these technical advancements, recovery of massive amounts of RNA from intracellular bacteria continues being a technical challenge due to two main factors. First, the low ratio of bacterial to eukaryotic RNA in the sample, ~0.1 picogram of RNA per bacteria cell compared to 10–20 picograms of RNA per eukaryotic cell (Westermann et al., 2012). Second, the short life of bacterial RNAs (Selinger et al., 2003). These features force in most cases to scale-up the infection assay in terms of the number of eukaryotic cells to be infected. As a representative example, ~ 3 × 10^7^ fibroblasts and a MOI of 50:1 (bacteria:cell) were infected with *S.* Typhimurium to obtain sufficient RNA for qRT-PCR assays (Ortega et al., 2012). In another study, ~ 6 × 10^7^ epithelial cells were infected at a MOI of 10:1 (bacteria: cell) with *L. monocytogenes* to obtain RNA for northern blotting assays (Quereda and Pucciarelli, 2014).

Other methods establish alternatives to the physical separation of “intact” intracellular bacteria from infected cells by focusing on selective enrichment of pathogen-derived sequences. An example is the method known as “selective capture of transcribed sequences” (SCOTS). This technique was first developed in the context of an infection model by two pioneer studies aimed to characterize genes expressed by *M. tuberculosis* and *S.* Typhimurium inside macrophages (Graham and Clark-Curtiss, 1999; Morrow et al., 1999). SCOTS is based on the capture of cDNA of pathogen-derived transcripts using biotinylated bacterial chromosomal DNA. This pathogen cDNA sample is prepared using the host and pathogen RNA mixture obtained from the infected tissue, organ or cell culture. In the initial studies, captured cDNAs were sequenced to identify genes expressed by intracellular bacteria or differentially expressed in two extracellular growth conditions Graham and Clark-Curtiss, 1999; Morrow et al., 1999; Daigle et al., 2001. In more recent studies, captured cDNAs are hybridized in whole-genome microarrays (Faucher et al., 2006, 2011; Emboule et al., 2009; Tolman and Valvano, 2012; Guo et al., 2014). SCOTS represents a useful method to obtain transcriptome data from intracellular bacteria avoiding the isolation of intact bacteria from host cells. SCOTS has also been shown to be useful for detecting small amounts of mRNA from low numbers of intracellular bacteria (Faucher et al., 2011). Noteworthy, despite the potential of SCOTS technology to analyze gene expression in intracellular bacteria, no study has yet used this method to determine expression of non-coding RNAs inside host cells. A probable reason might be that classical microarray technology based on oligonucleotides designed in the “coding genome” was rapidly replaced by tiling arrays and deep-sequencing technologies, which provide information on any type of RNA expressed on a defined condition. Although SCOTS is theoretically feasible using tiling arrays or deep sequencing, this combination has not been reported yet. There are also examples of sRNA expression profiling in intracellular *S.* Typhimurium using classical microarrays in which intergenic regions and a small number of sRNA sequences were represented (Ortega et al., 2012). Subsequent RNA-seq studies in this pathogen identified additional sRNA species (Kroger et al., 2012).

Taking into account these facts, investigators face two important questions when their objective is to determine gene expression profiling in intracellular bacteria. The first decision is whether or not to physically separate intracellular bacteria from host cell debris. These intact intracellular bacteria are precious material for any type of subsequent expression analyses, including tiling arrays, RNA-seq and individualized gene expression assays (e.g., qRT-PCR). The second decision is whether to directly obtain gene profiling data directly from the infected sample avoiding separation of bacterial and eukaryotic materials. In this latter case, the investigator could use SCOTS if pathogen information is the ultimate goal. There are also commercial kits that allow recovery of bacterial RNA from samples of infected eukaryotic cells containing host-bacterial RNA mixtures. These kits are based on a prior purification step that removes eukaryotic RNA (28 S, 18S rRNAs, and polyA^+^ mRNA), which is followed by selective capture of bacterial rRNA. A positive aspect of this procedure, as in the SCOTS method, is that no physical separation of intact intracellular bacteria is required since the procedure is performed on “total” (host-pathogen) RNA. Moreover, it guarantees that bacterial sRNAs remain in the sample. To date, no study has reported yet the usage of these kits for gene expression profiling in pathogens located inside eukaryotic cells, although they should be evaluated given its potential benefits. A comparative study examining the efficacy of protocols relying on bacteria isolated from infected host cells relative to those methods based on “total RNA” will be, undoubtedly, of extraordinary value. This type of studies might provide useful guidelines for future research involving global gene expression in intracellular bacteria.

Lastly, it should be noted the recent efforts aimed to analyze simultaneously gene expression in both host cells and intracellular bacteria. Technologies as “dual RNA-seq” allows to reach such goal (Westermann et al., 2012). These technologies rely on deep-sequencing of total RNA obtained from the infected culture. Further *in silico* analysis allows to identify eukaryotic and prokaryotic sequences and to assign them to both the host cell and the pathogen, respectively. Advantages of this method are that it does not require physical separation of the intracellular bacteria and that it avoids the usage of microarrays. The information relative to gene expression in the eukaryotic cells derives however from both infected and non-infected cells. This factor could be a major caveat in infection models in which the infection rate is relatively low, even when using a high MOI. Moreover, most infection models show that there is heterogeneity regarding the number of intracellular bacteria per host cell as the infection progresses. There is also evidence that intracellular bacteria can behave in a heterogeneous manner within the infected cell. For example, for *S.* Typhimurium it has been shown that actively growing and persistent bacteria co-exist in the same macrophage (Helaine et al., 2010). *S.* Typhimurium intracellular populations with distinct intracellular locations, cytosol vs. intravacuolar, have also been observed in epithelial cells (Knodler et al., 2010; Malik-Kale et al., 2012). Interestingly, these two populations are transcriptionally distinct, at least relative to virulence genes encoded in pathogenicity islands (Knodler et al., 2010). It is expected that RNA regulation may proceed also differently in these bacterial populations undergoing such distinct lifestyles inside the host cell. Considering these facts, it is tempting to think of future technologies able of combining the robustness of modern RNA-seq technologies with minimally-perturbing sorting procedures. Such cell separation methods could make use of reporters based on genes differentially expressed in infected vs. non-infected host cells or in the proliferating vs. non-proliferating intracellular bacterial populations.

## Perspective and challenges

Infection is a multidimensional complex event and several reasons provide a high degree of uncertainty when investigators try to model it. Thus, infection proceeds through distinct stages with different type of associated stresses. The pathogen also interacts during this journey with varied and complex systems, like the resident microbiota, the immune response, and the intracellular eukaryotic cell milieu. In addition, bacterial populations are not phenotypically homogeneous and display complex responses *in vivo* that might not be possible to model using regular genetic approaches *in vitro* (Diard et al., 2013). To analyze how infection is regulated (by non-coding RNAs or other regulators), it is necessary to discuss again how the infection process is approached and to integrate the bunch of information obtained in a carefully planned and systematic way.

For example, the study of intracellular bacterial pathogens needs a homogenization in the infection experimental set-ups. Infection protocols differ substantially from lab to lab even for the same pathogen: the way to prepare bacteria for infections, the MOI, the host cell type or animal model, and the protocols for sample preparation and gene expression analysis. Most times, there is not a scientific reason behind but it is just the “the way we do it here.” It would be interesting to reduce the experimental variability at this point since this is critical for investigations dealing with intracellular bacterial pathogens that have inherent technical difficulties.

Another important aspect highlighted in this review is the dynamics of the intracellular infection. Although a glance was obtained in a study with non-growing intracellular *S.* Typhimurium that showed varied expression of many sRNA along the infection (Ortega et al., 2012), we are still far from knowing many aspects of these alterations in this and many other pathogens. The field of RNA regulation should deal with this variable, although it clearly represents a major experimental challenge for which no magic solution currently exists. For example, a technology analogous to SmartFlare™, which allows real-time monitoring of gene expression in eukaryotic cells, is needed to address these dynamics-related questions on the bacterial side. This approach will nonetheless be biased to those genes of interest for the investigator. As a consequence, a “global picture” of the dynamics in the sense of the regulation exerted simultaneously by many sRNAs in intracellular bacteria seems still far for obtaining in a short-term basis. The visualization “over time” of both sRNA and targets might allow us to eventually define an “*sRNA-regulon*” once we know which sRNAs are regulating which transcripts or proteins.

The valuable “dual” gene expression analyses existing to date (Westermann et al., 2012; Humphrys et al., 2013) are clearly a direction that the field should boost. In addition, combination of sequencing data with sRNA target prediction algorithms could be helpful to reveal new RNA regulatory circuits (Wright et al., 2013; Kery et al., 2014). For example, using a similar strategy to that of the transcription factor analysis (Haverty et al., 2004), the dynamic expression of transcripts, proteins and sRNA regulators could also be integrated computationally. Other aspects to be technically intensified and improved include the proteomics applied to intracellular bacteria, which still has few representative studies. Cell sorting methods capable of separating the distinct host cell and bacterial populations co-existing in the *in vitro* infection model are also urgently needed.

Lastly, recent original studies have suggested a probable action of bacterial sRNAs in eukaryotic cells. Thus, the production of the sRNAs OxyS and DrsA in *E. coli* cells used to feed *Caenorhabditis elegans* was shown to interfere expression of worm genes related to chemosensory behavior and lipid metabolism (Liu et al., 2012). In another study, deep-sequencing uncovered an unexpected large number of non-coding RNAs in the plant pathogen *Agrobacterium tumefaciens* (Wilms et al., 2012), known to transfer the Ti-plasmid to the plant cell by a specialized type IV-secretion system (T4SS). It was hypothesized that some of these sRNAs could interfere with host physiology. Intriguingly, some bacterial pathogens that infect mammalian cells such as *Bartonella* spp, *Rickettsia* spp., *Brucella* spp., and *Helicobacter pylori*, also encode T4SS and evidence for DNA transfer to the eukaryotic cell has been found for some of them (Llosa et al., 2012; Fernandez-Gonzalez and Backert, 2014). Altogether, these observations support the tempting idea of some non-coding RNAs being transferred by bacterial pathogens to the host cell as part of the intracellular infection process. Such hypothetical sRNA transfer could occur by an active mechanism or, indirectly, as material released from some cells of the intracellular bacterial populations that naturally undergo death and lysis within the infected host cell. This latter assumption takes into account biotechnological studies designed to successfully transfer DNA to the eukaryotic cell using *L. monocytogenes* and *S.* Typhimurium engineered to undergo lysis upon entry into the host cell (Kuo et al., 2009; Kong et al., 2012). With no doubt, trans-kingdom action of bacterial sRNAs might open a fascinating new area in the field of RNA regulation but a definitive proof sustaining this hypothetical sRNA transfer phenomenon is still needed.

### Conflict of interest statement

The authors declare that the research was conducted in the absence of any commercial or financial relationships that could be construed as a potential conflict of interest.
